# Supplementary material for: Serum Concentrations of Selected Organochlorines in Pregnant Women and Associations with Pregnancy Outcomes. A Cross-Sectional Study from Two Rural Settings in Cambodia
Source: Int J Environ Res Public Health. 2020 Oct 20;17(20):7652. doi: 10.3390/ijerph17207652 (PMC7589876; doi:10.3390/ijerph17207652)
Supplement: Supplementary file 1 [file ijerph-17-07652-s001.pdf]

Table S1. Blood levels (wet weight picogram/microliter) of polychlorinated biphenyls (PCBs), hexachlorocyclohexane (HCH) and organochlorine pesticides (OCPs) in the inland and floating area.

* Geometric mean with 95% confidence intervals.

POPs	Inland Area (n = 120)				Floating Area (n = 74)			
	GM*	95% CI*	Median	Min-Max	GM*	95% CI*	Median	Min-Max
**PCB 52**	0.010	0.010-0.010	0.010	0.010-0.010	0.010	0.010-0.010	0.010	0.010-0.010
**PCB 99**	0.008	0.008-0.008	0.008	0.008-0.055	0.008	0.008-0.008	0.008	0.008-0.008
**PCB 101**	0.016	0.016-0.016	0.016	0.016-0.020	0.016	0.016-0.016	0.016	0.016-0.016
**PCB 118**	0.018	0.017-0.019	0.015	0.015-0.112	0.018	0.017-0.020	0.015	0.015-0.048
**PCB 138**	0.015	0.014-0.017	0.012	0.012-0.601	0.016	0.014-0.018	0.012	0.012-0.077
**PCB 153**	0.022	0.019-0.025	0.014	0.014-1.170	0.020	0.017-0.022	0.014	0.014-0.073
**PCB 180**	0.029	0.028-0.030	0.028	0.028-0.223	0.029	0.027-0.032	0.028	0.028-0.423
**α-HCH**	0.043	0.043-0.043	0.043	0.043-0.043	0.043	0.043-0.043	0.043	0.043-0.043
**β-HCH**	0.103	0.103-0.103	0.103	0.103-0.103	0.103	0.103-0.103	0.103	0.103-0.103
**γ-HCH**	0.053	0.053-0.053	0.053	0.053-0.053	0.053	0.053-0.053	0.053	0.053-0.053
**δ-HCH**	0.035	0.034-0.035	0.034	0.034-0.070	0.034	0.034-0.034	0.034	0.034-0.034
**HCB**	0.039	0.037-0.042	0.040	0.010-0.093	0.058	0.051-0.065	0.054	0.011-0.196
**Heptachlor**	0.137	0.134-0.140	0.135	0.135-0.352	0.135	0.135-0.135	0.135	0.135-0.135
**Heptachlor epoxide**	0.069	0.069-0.069	0.069	0.069-0.069	0.069	0.069-0.069	0.069	0.069-0.069
**o,p-DDE**	0.008	0.008-0.008	0.008	0.008-0.010	0.008	0.008-0.008	0.008	0.008-0.008
**p,p'-DDE**	0.627	0.527-0.747	0.579	0.020-4.760	0.975	0.811-1.170	0.869	0.270-8.050
**o,p-DDD**	0.007	0.007-0.008	0.007	0.007-0.018	0.007	0.007-0.007	0.007	0.007-0.007
**p,p'-DDD**	0.008	0.008-0.008	0.008	0.008-0.010	0.008	0.008-0.008	0.008	0.008-0.008
**o,p-DDT**	0.020	0.020-0.020	0.020	0.020-0.020	0.020	0.020-0.020	0.020	0.020-0.020
**p,p'-DDT**	0.039	0.036-0.042	0.033	0.030-0.519	0.038	0.034-0.042	0.033	0.033-0.401
**Mirex**	0.022	0.022-0.023	0.022	0.020-0.113	0.023	0.022-0.024	0.022	0.022-0.061
**Aldrin**	0.052	0.052-0.052	0.052	0.050-0.052	0.052	0.052-0.052	0.052	0.052-0.052

The Questionnaires

The subject volunteered to participate in this study and agreed that we extract necessary information from their hospital records after delivery.

If you have questions or need more information, please do not

hesitate to call To …………………………………………………..

Subject ID: …………………….

Area Code: ……………………. Health Center: ……………………

SECTION A: MATERNAL SOCIOECONOMIC INFORMATION

1. Patients name: …………………. Age:…………….

2. Address:

……………Village…………………Commune…………District:……………….

Telephone number:…………………

3. Gravida....................Para:………………………

4. Education ………………………Occupation ………………………..

5. Husband? ❑ Death ❑ A live ➲ If alive how old is he?:……………

➲ Education.......................... ➲ Occupation...............................

6. Major illnesses of mother, ❑ No ❑ Yes, Specify…………………………………

7. Medication of mother if daily, ❑ No ❑ Yes, Specify…………………………………

8. How many children do you already have?

➲ Baby Girl: ...., Age……………………………………….

➲ Baby Boy: .... Age……………………………………….

➲ Stillborn?:……………………..

➲ Died under five?:…………………………..

9. Breastfeeding of previous babies, ❑ No ❑ Yes, if yes how long…………………

10. How long have you lived at present address: Years:…………………………….

11. How do you describe your area of residence? ❑ Urban ❑ Rural

12. Sources of drinking water? ❑ Tap ❑ Lake ❑ Rain water ❑ Borehole ❑ Bottle

Other, specify:……………………………………………………………………………..

13. Do you use pesticides ❑ No ❑ Yes, if yes where? ❑ Inside home ❑ Rice Field ❑ On farm/garden

14. Do you or member of family fish? ❑ No ❑ Yes, if yes where?

42 ❑ Lake ❑ Stream ❑ Large River

SECTION B: DIET AND LIFESTYLE
Daily food sources of mother

Type of Food	Description				Frequency								
	**Medium serving size**	**Small**	**Medium**	**Large**	**Never**	**1/month**	**2-3 month**	**1/week**	**2/week**	**3-4/week**	5-6/week	1/day	2+/day
Meat													
Beef	100g												
Pork	100g												
Chicken	200g												
Fish													
Fresh Fish	100g												
Dry salty Fish	50g												
Smoke Fish	50g												
Vegetable													
Salad	50g												
Cabages	100g												
Spinages	100g												
water convolvulus	50g												
Cucomber	50g												
Eggplants	50g												
luffa gourds	50g												
Pumkins	50g												
Moringa	50g												
Beans	50g												
Bean Sprouse	50g												
Egg	1												
Papaya	100												
Others													
Rice	100g												
Soya Bean	50g												
Mung Beans	5og												

Sweat Potatoe	1												
Sugar Can Juice	1 cup												
Corn	1												
Coconut	1												
Banana	1												
Mango	1												
Pinapple	1												
Milk	1 can												

15. Sources of food: ❑ Own/local production ❑ Local market
❑ Supermarket ❑ Imported

Lifestyle of mother

➲ Do you smoke? ❑ No
❑ Yes, ➪If yes how many cigarettes daily…………………

➲ Do you drink alcohol
❑ No
❑ Yes, ➪ If yes what kind……………………
➪ How many tots daily…………………

SECTON C: MATERNAL INFORMATION POST DELIVERY MEDICAL INFORMATION

To be completed by attending Health Center Staff or designated field worker.

1. Maternal weight before delivery: ….. Kilograms ………….
2. Maternal height: ………………………cm……………………….
3. Previous spontaneous abortions 1. trimester: (if available)………..
   1 at how many months
   2 at how many months
   3 at how many months

4. Previous spontaneous abortions 2. trimester (if available)………
   1 at how many months
   2 at how many months
   3 at how many months
5. Previous preterm deliveries <week 37 (if available)……………
   1 at how many months
   2 at how many months
   3 at how many months
6. Any infertility problems –time to pregnancy – if any:
   ………………………………………………………………………………
7. Any complication/problems during pregnancy (hypertension, pre-eclampsia,
   infections)…………………………………………………………………
   ……..

SECTION D: INFORMATION ABOUT THE NEWBORN CHILD

1. Mode of delivery: ❑ Nornal Deliver ❑ Vacum ❑ CS

2. Birth weight of baby: ………………kg…..
3. Birth length of baby: …………………cm
4. Head circumference of baby: …………cm
5. Gestation age of baby (based on Naegele term):
6. APGAR score 1min………
   5min……… Any sign of
   asphyxia? ❑ No
   ❑ Yes

7. Gender of baby……………
8. Congenital malformations (visible at birth) ❑ No ❑ Yes
9. Any other medical observations or conditions
   ❑ No ❑ Yes, Specify ....……………………
